# Patterned growth of InGaN/GaN quantum wells on freestanding GaN grating by molecular beam epitaxy

**DOI:** 10.1186/1556-276X-6-117

**Published:** 2011-02-04

**Authors:** Yongjin Wang, Fangren Hu, Kazuhiro Hane

**Affiliations:** 1 Department of Nanomechanics, Tohoku University, Sendai 980-8579, Japan

## Abstract

We report here the epitaxial growth of InGaN/GaN quantum wells on freestanding GaN gratings by molecular beam epitaxy (MBE). Various GaN gratings are defined by electron beam lithography and realized on GaN-on-silicon substrate by fast atom beam etching. Silicon substrate beneath GaN grating region is removed from the backside to form freestanding GaN gratings, and the patterned growth is subsequently performed on the prepared GaN template by MBE. The selective growth takes place with the assistance of nanoscale GaN gratings and depends on the grating period *P *and the grating width *W*. Importantly, coalescences between two side facets are realized to generate epitaxial gratings with triangular section. Thin epitaxial gratings produce the promising photoluminescence performance. This work provides a feasible way for further GaN-based integrated optics devices by a combination of GaN micromachining and epitaxial growth on a GaN-on-silicon substrate.

PACS

81.05.Ea; 81.65.Cf; 81.15.Hi.

## Introduction

It's of significant interest to conduct the fundamental research as well as the applied study on the epitaxial growth on patterned GaN-on-silicon substrate [1-9]. Commercial GaN-on-silicon substrates make this research feasible [10], and novel epitaxial structures can be generated with smooth facets and are free of etching damage. It can also provide a great potential for further integrated GaN optics devices by a combination of the epitaxial growth, etching of GaN and silicon micromachining.

Compared to other growth techniques, the selective growth of GaN by molecular beam epitaxy (MBE) is relative difficult [11,12]. The substrate also impacts on the epitaxial growth. As the epitaxial growth of GaN on patterned Si or SiO_2 _substrates, GaN nanocolumns are easily formed due to random nucleation [13,14]. Selective area growth of GaN can produce periodic GaN nanocolumns with the assistance of nanostructured Ti-mask [15,16]. Recently, the selective growth of GaN by MBE is realized on patterned GaN-on-silicon substrate without introducing additional dielectric mask [17]. The shape and the growth area have the dominant influence on the realization of the selective growth by MBE. This approach enables easy fabrication and scaling, opening the great potential for a large variety of novel GaN-based devices.

In this study, we extend our research on the patterned growth of InGaN/GaN quantum wells (QWs) on freestanding nanoscale GaN gratings by MBE. Various freestanding GaN gratings are processed on a GaN-on-silicon substrate by a combination of electron beam (EB) lithography, fast atom beam (FAB) etching of GaN, and deep reactive ion etching (DRIE) of silicon. The patterned growth by MBE is performed on the prepared GaN template. Through the introduction of nanoscale grating structures, the selective growth occurs and depends on the grating period and the grating width. The optical performances of the resultant epitaxial gratings are characterized in photoluminescence measurements.

## Fabrication

**Figure 1:**
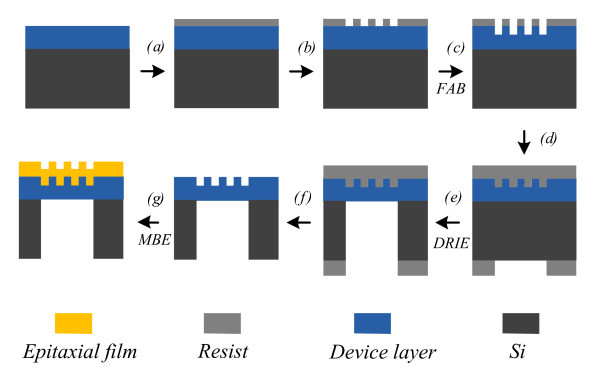
**Schematical process of patterned growth on freestanding GaN grating by MBE**.

The proposed epitaxial growth of freestanding GaN grating is implemented on GaN-on-silicon substrate, consisting of 280 nm GaN layer, 450 nm Al_*x*_Ga_1 _- _*x*_N layer (0.70 to approximately 0.20 Al mole fraction), 200-nm AlN buffer layer and 200-μm silicon handle layer. The fabrication process, described in detail elsewhere [17-19], is schematically illustrated in Figure 1. Nanoscale gratings are patterned in ZEP520A resist using EB lithography, and the resist structures act as a mask for FAB etching of GaN (steps a-b). The Cl_2 _gas is used as the process gas, and the etching depth is about 200 nm (step c). Then the residual EB resist is stripped and the processed device layer is protected by thick photoresist (step d). Silicon substrate beneath the GaN grating region is patterned from backside and etched down to the AlN layer by DRIE, where the AlN layer serves as a definite etch stop (step e). The freestanding GaN gratings are generated by removing the residual photoresist and cleaned for the epitaxial growth (step f). The epitaxial growth is conducted on the processed GaN template by MBE with radio frequency nitrogen plasma as gas source (step g). The epitaxial films with a designed thickness of approximately 420 nm incorporate approximately 140-nm low-temperature buffer layer, approximately 200-nm high-temperature GaN layer, six-pair 3-nm InGaN/9-nm GaN QWs layer and 10-nm GaN top layer. The growth process is described below.

The patterned template is put into a high vacuum chamber and cleaned at the temperature of 280°C for 12 h. Then the template is transferred into the growth chamber and cleaned at the temperature of 800°C for 60 min. A 140-nm-thick buffer layer is deposited at the temperature of 700°C, and a 200-nm high-temperature GaN layer is then grown at the temperature of 780°C. The six-pair 3 nm InGaN/9 nm GaN MQWs is subsequently deposited at the temperature of 620 to approximately 640°C. Finally, a 10-nm GaN layer is grown at the temperature of 620°C.

## Experimental results and discussion

**Figure 2:**
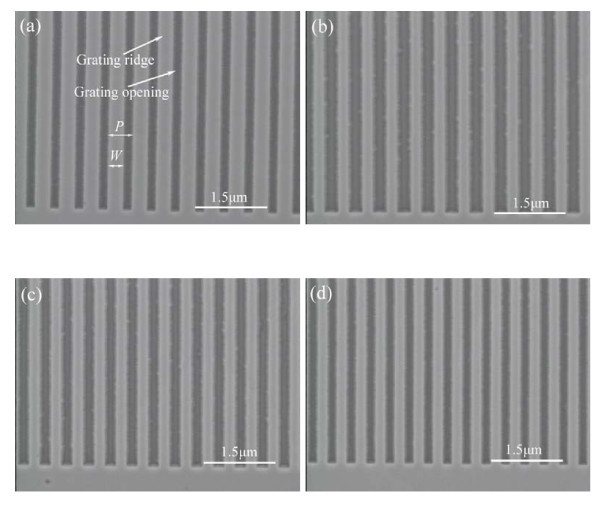
**SEMimages of GaN grating templates for the epitaxial growth of GaN**. **(a) **500-nm period, 300-nm-wide grating; **(b) **500-nm period, 200-nm-wide grating; **(c) **450-nm period, 200-nm-wide grating; **(d) **400-nm period, 200-nm wide grating.

Various freestanding GaN gratings are fabricated on a GaN-on-silicon substrate by a combination of EB lithography, FAB etching of GaN and DRIE of silicon [20]. Figure 2 illustrates scanning electron microscope (SEM) images of fabricated freestanding GaN gratings. The grating period and the grating width are expressed by *P *and *W*, as shown in Figure 2a, where *P *is 500 nm and *W *is approximately 300 nm. One period grating consists of the grating ridge and the grating opening. The GaN gratings illustrated in Figure 2b,c,d, have the same grating width of approximately 200 nm and have different grating periods of 500, 450, and 400 nm, respectively. The variation in the grating width *W *means the different distributions between the grating ridge and the grating opening, which plays an important role in the epitaxial growth.

**Figure 3:**
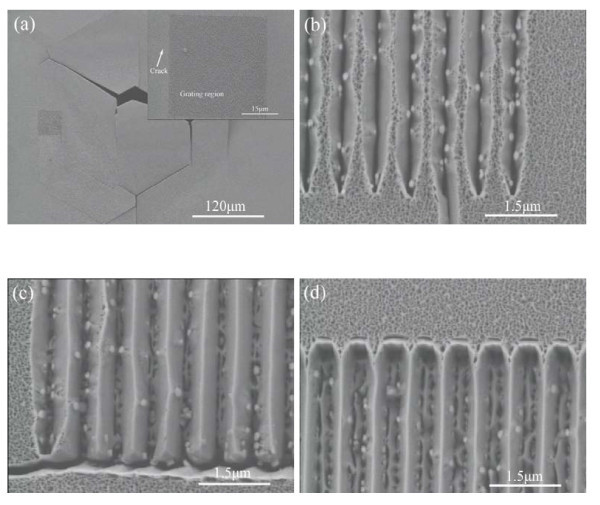
**Fracture related problems and epitaxial structures**. **(a) **Epitaxial grating on freestanding GaN membrane, and the *inset *is the zoom-in view of grating region; **(b)**, **(c) **and **(d) **the resultant 700-nm period epitaxial gratings: *(b) *500-nm-wide grating; *(c) *350-nm-wide grating; *(d) *250-nm-wide grating.

The built-in residual stress in GaN thin film on silicon substrate, which is due to the lattice mismatch and the thermal expansion coefficient mismatch, can result in the deflection problems for freestanding GaN membrane [21]. Although thin GaN membrane can guarantee sufficient stiffness for the fabrication of freestanding gratings during DRIE of silicon process, the fracture-related problems are shown in Figure 3a are evident in the freestanding GaN membrane after the epitaxial growth of GaN. These problems might be solved by adjusting the fabrication process. In order to avoid the damage to GaN gratings, the devices are not designed in the centre of the freestanding GaN membrane. The crack networks, which are caused by the lattice mismatch in the epitaxial layers, are observed on unpatterned GaN substrate, as illustrated in the inset of Figure 3a[22]. The crack does not occur in the GaN grating region, indicating the GaN gratings can compensate the lattice mismatch.

Figure 3b,c,d show the epitaxial structures on the 700-nm-period GaN gratings with the grating width *W *of approximately 500, approximately 350, and approximately 250-nm, respectively. Compared with unpatterned GaN substrate, grating structures locally change the diffusion conditions of adatoms from neighboring areas. Coherent growth is suppressed, and the selective growth takes place on the grating ridge with a preferential growth process on the low-energy side $\left\{10\bar{1}1\right\}$ facets. As the grating width *W *decreases, the area of the grating ridge is reduced. Thus, the surface diffusion can be sufficiently enhanced, resulting in complete coalescence between two side facets. Epitaxial gratings with smooth facets are observed in Figure 3c,d. Especially, Figure 3d demonstrates that the selective growth can also occur in the grating openings. Compared with Figure 3b, it can be concluded that a critical growth area is needed for the selective growth. When the growth area is too small, the selective growth is suppressed. On the other hand, it's difficult to complete the selective growth if the growth area is too large. The critical growth area might be dependent on the surface diffusion, which could be improved by adjusting the grating parameters.

**Figure 4:**
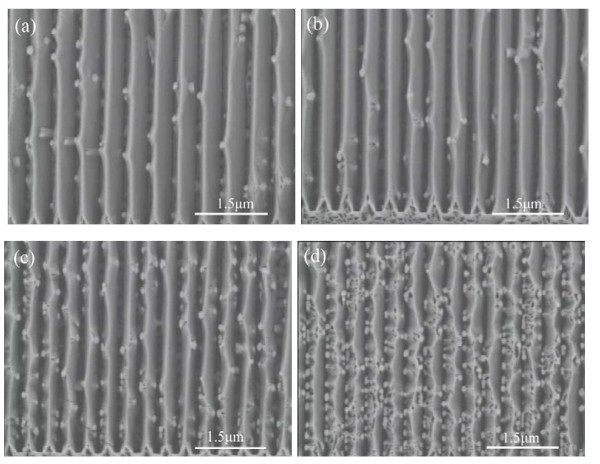
**SEM images of the resultant epitaxial gratings**. **(a) **500-nm period, 200-nm-wide grating; **(b) **450-nm period, 200-nm-wide grating; **(c) **400-nm period, 150-nm-wide grating; **(d) **400-nm period, 250-nm-wide grating.

In order to be more specific, we focus our attention on the epitaxial structures grown on the grating ridge. According to the above analysis, small grating period and small grating width are helpful for improving the surface diffusion to realize the selective growth on the grating ridge. On the other hand, nanoscale grating with small grating width is difficult to fabricate. Figure 4a, b shows the epitaxial gratings on the 200-nm-wide GaN grating with the grating periods of 500 and 450 nm, respectively. Coalescences between two side facets are completed for these epitaxial gratings, and side $\left\{10\bar{1}1\right\}$ facets are smooth with random GaN nanocolumns. The epitaxial structures on the 400-nm-period GaN gratings with the grating width *W *of approximately 150 nm and approximately 250 nm are illustrated in Figure 4c, d, respectively. The winding of GaN strip is found, which can be attributed the local fluctuation in the growth process. The number of epitaxial nanocolumns is increased, especially for 250-nm-wide GaN grating.

**Figure 5:**
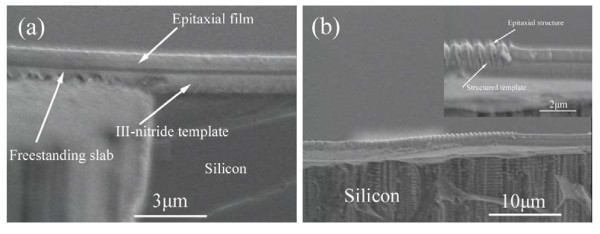
**Shape and the cross section of the epitaxial films**. **(a) **The cross section of the epitaxial films; **(b) **freestanding epitaxial grating structures, and the inset is the zoom-in view of grating region.

The shape and the cross section of the epitaxial films are shown in Figure 5. Since the sample is currently used for the development of backside thinning technique by wet etching of Al-based compounds, some freestanding epitaxial slabs are damaged in the wet etching process. The measured thickness of epitaxial films is about 510 nm, a little larger than the estimated thickness of approximately 420 nm. The freestanding III-nitride slab is deflected due to the residual stress, and the slab is thinner than that on silicon substrate, as shown in Figure 5a. One cross-section image of epitaxial grating is illustrated in Figure 5b. The inset is the zoom-in image of epitaxial grating, and the shape changes are clearly observed on different templates.

**Figure 6:**
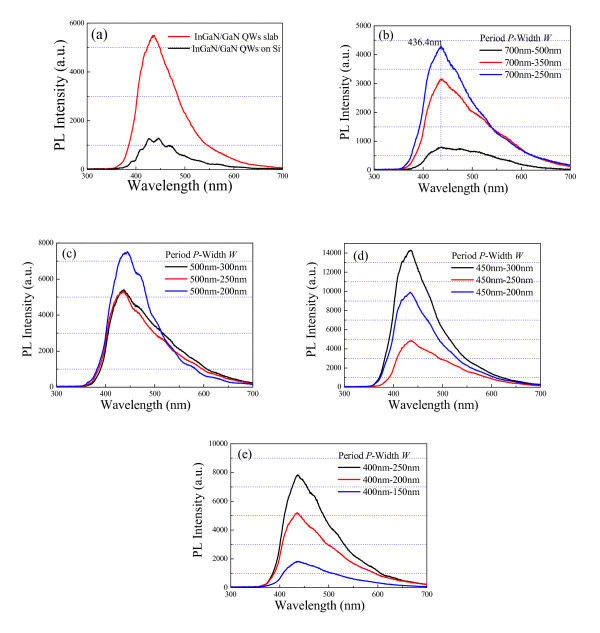
**Photoluminescence (PL) spectra of the resultant epitaxial gratings**. **(a) **PL spectra of epitaxial films on unpatterned template; **(b)**-**(e) **PL spectra of the resultant epitaxial gratings: *(b) *700-nm-period gratings; *(c) *500-nm-period gratings; *(d) *450-nm-period gratings; *(e) *400-nm-period gratings.

The photoluminescence (PL) spectra of the resultant epitaxial gratings are measured at room temperature using a 325-nm He-Cd laser source. The PL of InGaN/GaN QWs deposited on unpatterned area is shown in Figure 6a. Since the silicon substrate is removed and the slab is thinned by wet etching, the PL intensity is greatly for freestanding InGaN/GaN QWs slab. Figure 6b shows the PL spectra of 700-nm-period epitaxial gratings with various grating widths. The PL peaks at approximately 436.4 nm are associated with the excitation of the InGaN/GaN QWs active layers. With decreasing grating width *W *from approximately 500 nm to approximately 250 nm, the PL peak and the integrated intensity are significantly increased, corresponding to the improvement in the selective growth. The PL spectra of 500-nm-period epitaxial gratings are shown in Figure 6c and demonstrate the similar optical performances. The PL peaks are about 436.4 nm, and the corresponding PL intensities are improved, indicating that small grating period is helpful for the patterned growth. However, the PL spectra illustrated in Figure 6e, f is different as the grating period decreases to 450 and 400 nm, where the number of GaN nanocolumns is gradually increased. Especially for the 400-nm-period epitaxial gratings, the PL peaks are about 436.4 nm, but the PL intensities are greatly improved with increasing the grating width from approximately 150 nm to approximately 250 nm. However, the PL from 200-nm grating width sample is stronger than it from 250-nm-grating width sample for the 450-nm-period epitaxial gratings. It might be explained by the formation of epitaxial nanocolumns. Both epitaxial grating and nanocolumns contribute to the PL excitation. The number of epitaxial nanocolumns is increased with increasing the grating width, whereas the epitaxial gratings with smooth facets are easily formed with decreasing the grating width. Hence, the epitaxial structures generated in reality determine which one plays the dominant influence on the PL spectra. On the other hand, thin InGaN/GaN QWs layers are incorporated in the upper part of the epitaxial gratings, the film structures beneath smooth side facets are rough, and the scattering losses are thus very large. Consequently, there is no clear signal to reflect the interaction between the excited light and the grating structures.

## Conclusions

In summary, various freestanding GaN gratings are fabricated on a GaN-on-silicon substrate by a combination of EB lithography, FAB etching of GaN and DRIE of silicon. The patterned growth of InGaN/GaN QWs is performed on the processed GaN template by MBE. Nanoscale grating structures locally change the diffusion conditions of adatoms from neighboring areas, and the selective growth takes place with a preferential growth process on the low-energy side facets. Coalescences between two side facets are achieved to generate epitaxial gratings with triangular section, and the patterned growth depends on the grating period *P *and the grating width *W*. Thin epitaxial gratings produce the promising photoluminescence performance. This work provides a feasible way for further GaN-based integrated optics devices by a combination of GaN micromachining and MBE growth on a GaN-on-silicon substrate.

## Competing interests

The authors declare that they have no competing interests.

## Authors' contributions

YW carried out the device design and fabrication, performed the optical measurements, and drafted the manuscript. FH carried out the MBE growth. KH conceived of the study, and participated in its design and coordination. All authors read and approved the final manuscript.
